# Psychometric evaluation of the Polish adaptation of the Body Appreciation Scale-2 for Children (BAS-2C)

**DOI:** 10.1371/journal.pone.0309945

**Published:** 2024-09-12

**Authors:** Karolina Kubicka, Agata Trzcińska, Małgorzata Gambin

**Affiliations:** Faculty of Psychology, University of Warsaw, Warsaw, Poland; Tabriz University of Medical Sciences, ISLAMIC REPUBLIC OF IRAN

## Abstract

Positive body image is crucial in fostering self-esteem, mental well-being, and positive social functioning. However, our understanding of its development and role in school-aged children remains limited due to a scarcity of measures available for this age group across various countries and cultures. To bridge this gap, the aim of this study was to adapt the Body Appreciation Scale-2 for Children (BAS-2C) for Polish children aged 8–13 years. A total of 206 children completed BAS-2C to measure positive body image, the Figures Rating Scale to measure body satisfaction, and the Self-Perception Profile for Children to measure self-esteem. Parents provided information about their child’s weight and height, which enabled analysis of a child’s BMI. The results showed that the Polish version of BAS-2C exhibited a unidimensional factor structure, invariance across sex and age groups, and also provided evidence of very good internal consistency, test-retest reliability, and construct validity. BAS-2C scores were correlated positively with Figures Rating Scale, physical appearance and global self-esteem and were related negatively to age and BMI index, and weight group. Moreover, our research indicates that in older children, there are stronger correlations between positive body image and both global and physical appearance self-esteem, compared to younger children. The Polish version of BAS-2C has excellent psychometric properties and is appropriate for use with children aged 8–13 years.

## Introduction

A positive body image, a key component of the broader construct of body image, has been variously defined by different scholars (e.g. [1–3]). According to Tylka & Wood-Barcalow [2], positive body image focuses on (1) a positive opinion of the body, (2) respect for the body, (3) gratitude toward the body, (4) rejection of societal ideals of attractiveness, (4) inner positivity that affects outward appearance, (5) a broad conceptualization of beauty. Studies by Swami and collaborators [3] have shown that positive body image, particularly body esteem, significantly predicts all dimensions of well-being: emotional, psychological, and social in adults. Considerable body of research in adults and adolescents supports these theories and findings (e.g. [4–11]). However, little is known about the development of positive body image in children. Nevertheless, some findings in school-aged children are consistent with studies in adults and adolescents [12–14].

Several instruments were developed to measure positive body image in adults and adolescents: e.g. Experience of Embodiment [4] and The Body Appreciation Scale (BAS) [15]. A number of measures focus on examining body image and its various domains such as body shame, body image, and body satisfaction [5, 16, 17]. Nevertheless, there are fewer instruments to measure children’s perception of their bodies, regardless of the construct the researcher wishes to focus on. One such tool is the Figures Rating Scale [18], in which children first choose the picture which best resembles their appearance and then another that represents their desired look. Several newer versions of this scale, such as the Children’s Body Image Scale (CBIS) [19], were developed. There are also surveys for children that focus on appearance or weight [20].

To the best of our knowledge, the Body Appreciation Scale for Children (BAS-2C) [21] is the only instrument that measures positive body image in children. This scale, evolved from the Body Appreciation Scale (BAS) [2], is the most accurate and widely used instrument for measuring positive body image in adolescents and adults [22–24]. The version for children was originally developed for children aged nine to eleven in England [21], and was then adapted for use in Japanese and Portuguese linguistic contexts [13, 14]. The scales contain ten items and exhibit a one-factor structure, and satisfactory psychometric properties both for girls and boys across all samples [13, 14, 21].This research has also demonstrated a positive correlation between positive body image and self-esteem, life satisfaction, body esteem, healthy eating habits and a negative correlation with media influence and media pressure. Only two studies have explored the issue of measurement invariance of BAS2-C. The Japanese BAS-2C showed invariance between genders [14]. In contrast, the research conducted in Portugal identified a lack of invariance between sexes [23]. Further analysis of specific items revealed that item #9 ("I am comfortable in my body") functioned differently for girls and boys, indicating that this item does not measure body appreciation in the same way for male and female children. Surprisingly, among studies on the psychometric properties of BAS2-C, only the research conducted in England [21] identified gender differences in positive body image, with boys showing a more positive body image than girls. These findings are unexpected taking into account previous studies indicating that boys and men are more satisfied with their bodies compared to girls and women [e.g. 8, 25, 26]. These differences are commonly understood in the context of societal beauty standards to which children are exposed to from a young age [7]. Girls face higher expectations than boys to be attractive and to take care of themselves. Consequently, girls and women are more focused on their bodies and demand more from themselves in this context, leading to higher dissatisfaction compared to boys and men [7]. However, the Body Apperception Scale for children measures positive body image, as emphasized by the scale’s authors. While it represents a different construct, it is closely related to the construct of "body dissatisfaction" [2]. The lack of significant gender differences in findings using the BAS2-C may also stem from varying cultural influences that shape body image perceptions differently across societies and countries [7, 27], or it could be due to the research being conducted with children at a developmental stage where gender-specific attitudes towards body image may not yet be fully established [28].

Nonetheless, there is still limited knowledge about the risk and protective factors that play a role in development of positive body image in children [6]. Studies in various cultures and age groups would enable research enhancing our understanding of body image development across developmental stages and in different cultural contexts. It is acknowledged that older children and adolescents exhibit a greater degree of self-focus than their younger counterparts, particularly in terms of increased attention to their bodies and body appearance [29]. However, an emerging trend reveals that even younger children encounter eating-related issues and struggle with eating disorders which are directly associated with body image concerns [7, 28, 29]. In light of these observations, it is crucial to conduct research encompassing both younger school-aged children and their older peers transitioning into adolescence. This approach would facilitate the identification of pivotal transitional moments and the diagnosis of influential factors. Additionally, it would enhance understanding of the processes underlying the formation of body image, thereby paving the way for the implementation of more targeted and effective strategies for body image prevention and intervention.

Therefore, the purpose of this study was to adapt the Body Appreciation Scale for Children to the Polish cultural context and to 8–13 year old children broadening the age spectrum beyond the age groups engaged in prior research. Our objective was to expand the scale’s applicability across a broader age range beyond what the original scale authors had established [21]. This decision was aimed at enabling us to effectively capture shifts in the perception of positive body image that can occur during various stages of childhood development. Moreover, including younger children and those entering puberty or in early adolescence in the research could capture the moment when there is a "breakdown" in positive body image development. This could enable the design of appropriate interventions for children at this critical moment. Additionally, involving younger children in the research and examining their positive body image in relation to various factors could help to create preventive interventions that would sustain a positive body image during adolescence and adulthood. Expanding the age group for the use of this scale would facilitate longitudinal research to assess the effectiveness of these interventions. In the current study, for certain analyses, we categorized school-aged children into a younger group (8–10 years old) and an older group (11–13 years old), being either at the onset of or just entering the period of adolescence. This division allows for a more in-depth analysis of the changes in positive body image perceptions as children move from late childhood to the onset of adolescence.

To assess the psychometric properties, our study’s methodology involved conducting a confirmatory factor analysis (CFA) to explore the structural validity of the Polish version of the scale. We aimed to determine whether the scale exhibited a one-factor structure consistent with other versions of the scale [13, 14, 21] and investigate measurement invariance across gender and age groups. Additionally, our objectives encompassed establishing internal consistency and test-retest reliability for the adapted BAS-2C scale. Furthermore, we assessed construct validity through correlation analyses between the BAS-2C scale and variables such as self-esteem, BMI, and body satisfaction. We hypothesized that the BAS-2C scale would show negative correlations with BMI and weight-group (substandard, normal, above normal, and obesity), along with positive correlations with both global and physical appearance self-esteem, as well as body satisfaction. These hypotheses are grounded in previous research, where associations between these variables and body appreciation have been established in both children and adults [2, 13, 21, 30].

## Materials and methods

### Ethics statement

The study has received approval from the Research Ethics Committee at the Faculty of Psychology, University of Warsaw (Opinion no. 7/03/2021). Prior to the study, parents provided written informed consent for their child’s participation. Only children whose parents had given prior written informed consent were invited to participate in the study. Children provided verbal assent in line with the ethics committee guidelines.

### Participants and procedure

We employed the Mundfrom, Shaw, and Ke method [31] to determine the minimum sample size for Confirmatory Factor Analysis using a variables to factors (p/f) ratio of 10. A minimum sample size of 50 respondents was necessary to achieve excellent statistical power. However, in adherence to recommendations by other researchers (e.g., [32]), advocating for 10 respondents per item and a general rule of 100 participants per group for multi-group CFA [33, 34], we aimed to test 200 children, including a minimum of 100 boys and 100 girls. For the reliability test, the Sample Size Calculator by Arifin [35] was utilized, assuming an internal consistency coefficient of 0.75, with a minimum acceptable value of 0.50. Considering a power of 0.80 and a significance level of 0.05 (one-tailed), the required sample size was determined to be 36. To establish the validity of the BAS-2C, correlational analyses were planned. G*Power [36] software indicated that a sample size of 64 participants would be sufficient to detect medium effects at an alpha level of 0.05 and a power of 0.80.

A total of 206 children (106 girls and 100 boys) from a large city in Poland, aged between eight and thirteen years (M = 9.95, SD = 1.46) participated in the study. Children completed the questionnaires individually, with a paper-pencil method, being fully informed about the study’s objectives and having the freedom to halt their participation at any point. The recruitment for the study began on December 1, 2022, and ended on June 30, 2023. The first author of the manuscript collected the data and contacted parents, teachers and school head teachers. For children in the second and third grades, the researcher read questions, whereas older children independently read and completed the questionnaires. Parents filled out the information concerning the children and family at home and subsequently returned it in a sealed envelope.

Based on the BMI we calculated, the children were assigned to four weight groups: seven children (3.4%) to underweight (-1), 151 children (73.3%) to normal weight (0), 16 children (7.8%) to overweight (1), 17 children to (8.3%) obesity (2), 13 parents of the children (6.3%) did not respond to the question regarding their child’s weight and length, making it impossible for us to calculate the BMI. After 6 weeks 44 participants filled BAS-2C again to determine test-retest-reliability. The sociodemographic variables of the sample are shown in S1 Appendix.

### Measures

#### Body appreciation

The Body Appreciation Scale for Children (BAS-2C) [21] measures positive body image in children. The scale consists of ten items rated on a 5-point Likert scale (e.g., "I take a positive attitude towards my body"). The BAS-2C was adapted and translated into Polish according to guidelines for the translation of instruments in cross-cultural research [37]. This process entailed the execution of a high-quality translation, a blind back-translation, and the incorporation of inputs from Polish mental health professionals.

Furthermore, a pilot study involving children was conducted to assess the comprehensibility and applicability of the translated scale. In our pilot study, 8–10 year old children struggled with comprehension of the word “comfortable” in the context of the item #9. We explained to the children the meaning of this word, and then asked them for suggestions for a term that would be more comprehensible for their age group. All of them suggested the word “good”. This feedback, in conjunction with consultations with mental health professionals and our research team, led us to replace "comfortable" with "good".

The Cronbach’s alpha in the current sample was .91 for the entire sample, .89 for boys, and .92 for girls.

#### Body mass index (BMI)

Children’s BMI and weight-group were calculated based on height and weight (kg/m2) provided by parents. BMI was then converted to age- and sex-specific z-scores (BMIz) based on the Ola and Olaf standards [38] for children and adolescents in Poland, derived from the Using the centile calculator, we also assign children according to their BMI to a group: underweight (-1), normal weight (0), overweight (+1), obese (+2).

#### Self-esteem (physical appearance and global)

We utilized the Self-Perception Profile for Children [39], adapted for the Polish context [40], to assess children’s self-esteem. In our analyses, we exclusively employed the physical appearance (e.g. "Some kids think that they are good-looking BUT other kids think that they are not very good-looking") and global self-esteem (e.g. “Some kids are happy with themselves as a person BUT other kids are often not happy with themselves”) subscales, each consisting of 6 items. Higher scores in physical appearance indicate that a child is more happy with its own looks and physical characteristics such as height, weight, hair, and face. Higher scores in global self-esteem mean that a child has more general acceptance of oneself as a person, more likes oneself, and whether the child is more happy with its own life. Answers were rated on a scale from one to four. The Cronbach’s alpha in the current sample was .88 for physical appearance and .82 for the global self-esteem subscale.

#### Body satisfaction

In order to assess body satisfaction, the Figures Rating Scale [18] was utilized. This scale was created for children [18] and comprised seven female/male images that represented a spectrum of child body sizes, ranging from underweight (1) to obese (7). Children were requested to identify the image that bore the closest resemblance to their (1) own physique as well as their (2) ideal body figure. By calculating the discrepancy between the chosen "ideal" figure and the figure that most closely resembled the participant, body satisfaction scores are obtained, spanning from -6 to +6. A negative score indicates a desire to have a thinner body, while no discrepancy indicates contentment with one’s current body size. The scale exhibited good psychometric properties in the study of children aged 9–13 years [41]. Cronbach’s alpha was .73 in the current sample.

#### Data analytic strategy

To establish the factorial validity of the Polish version of the BAS-2C, confirmatory factor analysis (CFA) was conducted. As the normality assumptions were violated, the estimation method employed was a maximum likelihood with robust standard errors and Satorra-Bentler scaled test statistic (MLM) [42]. The analysis was performed using MPlus software version 7.2 [43]. According to the criteria outlined in the literature, we considered the following indicators as indicative of an acceptable model fit: χ2/df < 2, RMSEA < .08, CFI ≥ .90, TLI ≥ .90, and SRMR < 1 [44, 45]. The model fit was considered as good if RMSEA < .10, CFI ≥ .95, TLI ≥ .95 and SRMR < .08 [45–47]. In order to establish the correlation among errors we used a conservative approach. This involved examining modification indices (MI) that surpassed a threshold of 11 (χ2 0.999; (1) = 10.83), while also taking into account model saturation and goodness-of-fit measures. The interpretation of factor loadings followed the recommendations of Tabachnick and Fidell [48], where loadings equal to or greater than .32 were deemed acceptable.

To assess measurement invariance in the BAS-2C factor structure, initial single-group CFAs were conducted to separately examine the factorial structure of the BAS-2C in each group [49]. Gender stratification resulted in a sample of 106 cases for girls and 100 cases for boys. Regarding age groups, the sample was divided into two categories: 8–10 years old (younger group, n = 139) and 11–13 years old (older group, n = 67). Subsequently, multi-group CFA [50, 51] was performed using MPlus software to assess the consistency of the factor structure across gender and age categories. Factorial invariance is considered present when: 1) the construct is associated with the same set of items in each group (i.e., configural invariance), 2) the relations between the construct and the items, represented by factor loadings, show no significant differences across groups (i.e., metric invariance), and 3) both the factor pattern coefficients and intercepts are equivalent across groups (i.e., scalar invariance).

The internal consistency of the BAS-2C was evaluated using Cronbach’s alpha, with values greater than .70 considered satisfactory consistency. To assess the test-retest reliability over a period of four weeks, the intraclass correlation coefficient (ICC) was employed. ICC values of .75 or higher were considered excellent, values between .50 and .75 were considered moderate to good, values between .25 and .50 were considered fair, and values less than .25 were considered weak [31].

Construct validity was assessed using Spearman’s rho correlation coefficients and interpreted as follows: 0–0.30—no or weak correlation, .31 - .50—moderate to good, .51 - .70—strong, 0.71–1—very strong correlation. Gender variations in body appreciation were examined using the U Mann-Whitney test and evaluated based on Cohen’s [35] criteria: small effect size r = .10, moderate effect size r = .30, and large effect size r = .50.

## Results

### Descriptive statistics

The Kolmogorov-Smirnov test indicated that the distribution of the total BAS-2C scores was not normal (Z(206) = .15; p < .001). Independent samples U Mann-Whitney test results showed no significant differences in total BAS-2C scores between boys and girls, *U* = 5152.5, *z* =.-345, *p* = .730, *η*^*2*^ = .001. Descriptive statistics for Body Appreciation Scale-2 for Children (BAS-2C) items are presented in Table 1.

**Table 1:** Descriptive statistics for Body Appreciation Scale-2 for Children (BAS-2C) items.

BAS-2C items/ Polish version of BAS-2C items		M	SD	Skewness	Kurtosis
1.	I feel good about my body / Czuję się dobrze z moim ciałem	4.09	1.01	-.89	-.11
2.	I respect my body / Szanuję moje ciało	4.34	.90	-1.37	1.27
3.	I feel that my body has at least some good qualities / Czuję, że moje ciało ma przynajmniej trochę dobrych cech	4.13	.99	-1.11	.76
4.	I take a positive attitude towards my body / Mam pozytywne nastawienie do swojego ciała	4.17	1.04	-1.18	.69
5.	I pay attention to what my body needs / Zwracam uwagę na to, czego potrzebuje moje ciało i mój organizm	4.19	.99	-1.39	1.57
6.	I feel love for my body / Czuję, że kocham moje ciało	3.91	1.23	-.96	-.12
7.	I appreciate the different and unique things about my body / Doceniam różnorodne i wyjątkowe rzeczy w moim ciele	4.04	1.01	-.76	-.31
8.	You can tell I feel good about my body by the way I behave / Można poznać, że czuję się dobrze z moim ciałem po sposobie w jaki się zachowuję	3.78	1.16	-.69	-.37
9.	I am comfortable in my body / Czuję się dobrze w swoim ciele	4.32	.95	-1.55	2.24
10.	I feel like I am beautiful even if I am different from pictures and videos of attractive people (e.g., models/actresses/actors) / Czuję, że jestem piękna/y, nawet jeśli wyglądam inaczej niż atrakcyjni ludzie ze zdjęć i filmów (np. modele modelki, aktorzy/aktorki)	3.89	1.18	-.77	-.43
	**Total BAS-2C score**	40.85	.54	-1.14	.80
	**Total BAS-2C score (boys)**	40.98	7.21	-1.04	.86
	**Total BAS-2C score (girls)**	40.72	8.37	-1.18	.69

### Confirmatory factor analysis

**Fig 1:**
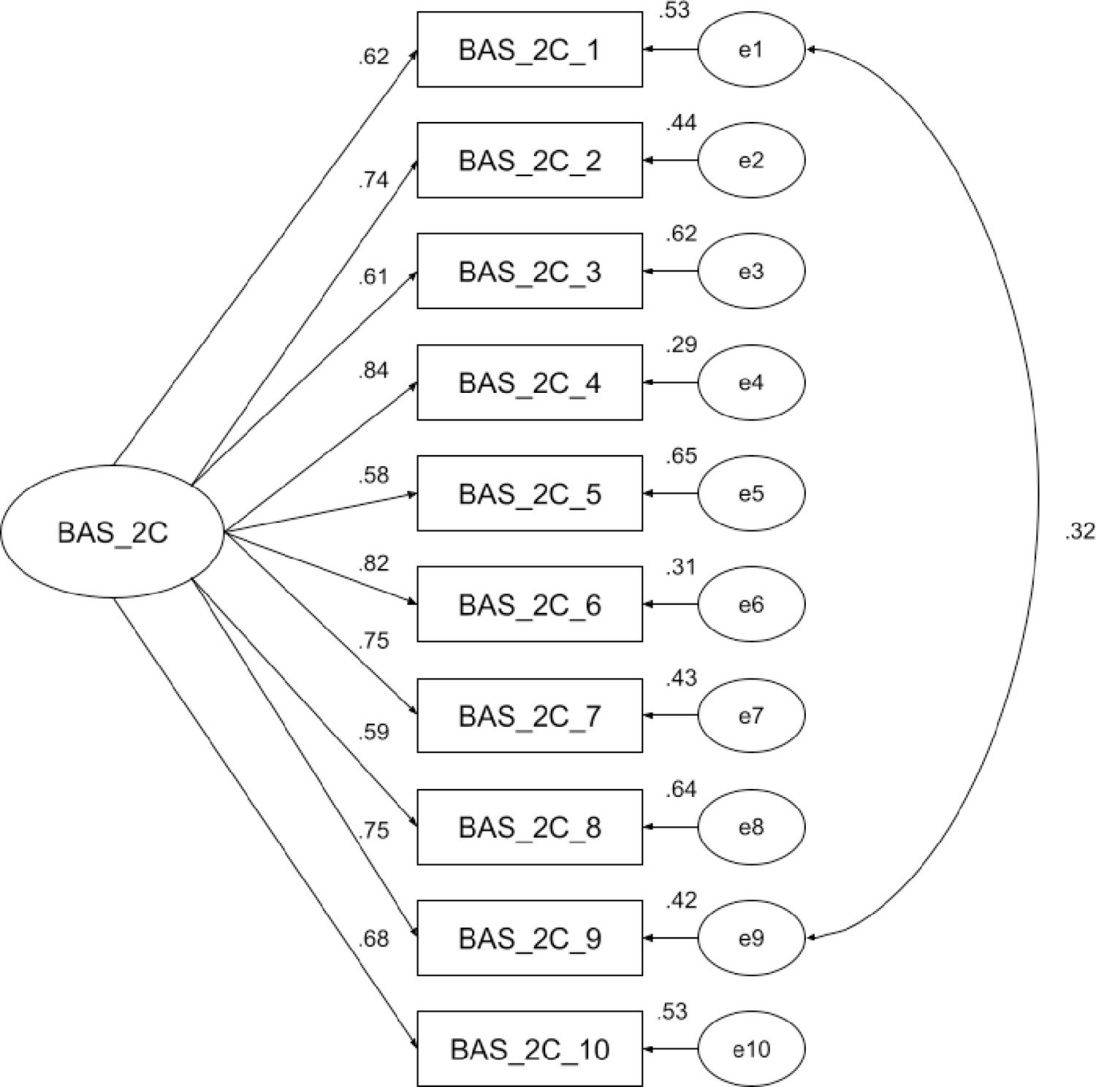
Path diagram and estimates for the one-dimensional model of the Body Appreciation Scale-2 for Children (BAS-2C). Note. All parameter estimates are standardized and significant at p < .001.

Previous studies [13, 14, 21] have shown that BAS-2C has a one-factor structure. We used confirmatory factor analysis (CFA) to examine whether this also holds true for the Polish adaptation. The analysis indicated that the model exhibited a good fit: χ2 (35) = 55.42, p = .015; χ2/df = 1.58; CFI = .974; TLI = .966; RMSEA = .053, 90% CI [.024, .079]; SRMR = .039. Item-factor loadings ranged from .58 (Item 5) to .84 (Item 4) and were deemed satisfactory. An examination of modification indices suggested a correlation between the error covariances of items 1 and 9 (MI = 13.79), resulting in an improved model fit: χ2 (34) = 43.09, p = .136; χ2/df = 1.27; CFI = .988; TLI = .984; RMSEA = .036, 90% CI [.000, .066]; SRMR = .033. Chi-Square Difference test (using the Satorra-Bentler Scaled χ2) indicated significant improvement of the model (TRd = 6.93, p < .01). Item-factor loadings ranged from .60 (Item 5) to .84 (Item 4) and were deemed satisfactory (Fig 1).

### Measurement invariance across gender and age groups

Initially, a modified model, incorporating the covariation between item 1 and 9, was assessed within each group utilized for invariance testing (Table 2). Findings substantiated the model’s validity across all subsamples extracted from the overall sample (boys, girls, younger children, and older children).

**Table 2:** Fit statistics of the BAS-2C model in each sample used for invariance testing.

Model	χ2 (df)	p	RMSEA	CFI	TLI	SRMR
Boys (n = 100)	54.44 (34)	.015	.078	.942	.924	.058
Girls (n = 106)	35.72 (34)	.387	.022	.996	.995	.035
Younger group (n = 139)	45.73 (34)	.086	.050	.966	.956	.052
Older group (n = 67)	30.81 (34)	.625	.000	1.00	1.10	.034

Subsequently, three dimensions (configural, metric, and scalar) of measurement invariance for the BAS-2C were assessed for gender and age groups. Concerning gender, the configural model exhibited a satisfactory fit to the data (Table 3), implying equivalent factor structures across gender categories. The χ2 changes for metric and scalar invariance models were statistically non-significant, and alterations in CFI and RMSEA were below the specified thresholds set by Rutkowski and Svetina [52] (cutoff values of .030 for CFI and .020 for RMSEA change). Therefore, measurement invariance for gender was confirmed.

**Table 3:** Model fit indices for gender and age group measurement invariance tests.

	Model Fit Indices					Model difference					
Model	χ2 (df)	p	RMSEA	CFI	SRMR	ΔM	Δχ2 (Δdf)	p	ΔRMSEA	ΔCFI	ΔSRMR
*Gender*											
M1: Configural	89.78 (68)	.040	.056	.974	.047	-	-	-	-	-	-
M2: Metric	101.65 (77)	.032	.056	.971	.079	M2 vs. M1	11.87 (9)	.221	.000	-.003	.032
M3: Scalar	109.23 (86)	.046	.051	.972	.081	M3 vs. M2	6.44 (9)	.695	-.005	.001	.002
*Age group*											
M1: Configural	78.67 (68)	.18	.039	.986	.047	-	-	-	-	-	-
M2: Metric	89.23 (77)	.16	.039	.984	.070	M2 vs. M1	10.56 (9)	.307	.000	-.002	.023
M3: Scalar	98.87 (86)	.16	.038	.983	.074	M3 vs. M2	9.54 (9)	.389	-.001	-.001	.004

Similarly, results from the measurement invariance tests for age groups indicated that the configural model adequately fit the data, signifying an equivalent factor structure of the BAS-2C across the two age groups (see Table 4). The χ2 changes for both metric and scalar invariance models were non-significant. Furthermore, the reductions in CFI and RMSEA changes were well below the predetermined thresholds (i.e., .030 for CFI and .020 for RMSEA). This suggests that the contribution of each item to the latent factor is comparable across both age groups, and differences in latent means reflect shared variances in observed items. Therefore, measurement invariance for both groups was confirmed.

**Table 4:** Descriptive statistics and correlations (Spearman’s rho) between body appreciation and other measures included in the study for the entire sample.

Variables	M(SD)	[1]	[2]	[4]	[5]	[6]	[7]	[8]
[1] Body appreciation	40.85 (7.82)	--						
[2] Age	9.95 (1.46)	-.28**	--					
[4] BMI	17.62 (3.28)	-.36**	.43**	--				
[5] Weight-group ^a^	.23 (.65)	-.28**	.21**	.58**	--			
[6] Physical appearance self-esteem	3.11 (.83)	.71**	-.28*	-.46**	-.34**	--		
[7] Global self-esteem	3.28 (.68)	.68**	-.34**	-.35**	-.24**	.73**	--	
[8] Body satisfaction	-.64 (1.12)	.34**	.06	-.48**	-.38**	.42**	.31**	--

* p < .05

** p < .01

^a^ Kendall’s tau correlation

### Internal consistency and test-retest reliability

Cronbach’s alphas for the BAS-2C were .91 for the entire sample, .89 for boys, .92 for girls, and .87 for younger group, .93 for older group, demonstrating excellent internal consistency of the Polish measure. To assess stability over time, the Intraclass Correlation Coefficient (ICC) was calculated. The ICC was found to be .92, p < .001 (95% CI: .85, .95) for the full sample, .86, p < .001 (95% CI: .64, .94) for boys, .93, p < .001 (95% CI: .83, .97) for girls and .85, p < .001 (95% CI .68, .93) for younger group, .91, p < .001 (.77, 97) for older group. These results indicate excellent test-retest reliability.

### Construct validity

The results aligned with our expectations, revealing a moderate negative correlation between the BAS-2C and BMI as well as weight-group. Additionally, the BAS-2C demonstrated strong positive correlations with global self-esteem and physical appearance, self-esteem, along with a moderate correlation with body satisfaction. We also observed a weak negative correlation between body appreciation and the child’s age (see Table 4).

The correlations, separated by gender, are shown in Table 5. Using Fisher’s r-to-z transformation, we found that the correlation coefficients between the BAS-2C and BMI (z = -0.24, p = .810), weight-group (z = -.92, p = .179), physical appearance (z = 1.36, p = .174), and body satisfaction (z = -0.08, p = .936) did not differ significantly between boys and girls. However, it is worth noting that the correlation between body appreciation and global self-esteem was higher for girls compared to boys (z = 2.53, p < .05). These results provide support for the construct validity of the Polish version of BAS-2C.

**Table 5:** Descriptive statistics and correlations (Spearman’s rho) between body appreciation and other measures included in the study for boys (upper diagonal) and girls (bottom diagonal).

Variables	Boys M(SD)	Girls M(SD)	[1]	[2]	[4]	[5]	[6]	[7]	[8]
[1] Body appreciation	40.98 (7.22)	40.73 (8.37)	--	-.23*	-.34**	-.21*	.64**	.58**	.35**
[2] Age	10.05 (1.46)	9.85 (1.46)	-.31**	--	.49**	.19*	-.25*	-.29**	.03
[4] BMI	18.06 (3.33)	17.23 (3.20)	-.37**	.38**	--	.60**	-.53**	-.35**	-.51**
[5] Weight-group ^a^	.29 (.66)	.17 (.64)	-.33**	.23**	.57**	--	-.38**	-.21*	-.47**
[6] Physical appearance self-esteem	3.09 (.78)	3.13 (.87)	.74**	-.29*	-.40**	-.30**	--	.66**	.50**
[7] Global self-esteem	3.32 (.61)	3.24 (.74)	.77**	-.39**	-.36**	-.28**	.79**	--	.35**
[8] Body satisfaction	-.68 (1.05)	-.60 (1.18)	.34**	.10	-.45**	-.31**	.36**	.28**	--

* p < .05

** p < .01

^a^ Kendall’s tau correlation

The correlations, separated by younger group (8–10 years) and older group (11–13 years), are shown in Table 6. In both younger and older children, we observed positive correlations between positive body image and several factors: physical appearance, global self-esteem, and body satisfaction. Additionally, there was a negative correlation between positive body image, weight group and BMI. Using Fisher’s r-to-z transformation, we found that the correlation coefficients between the BAS-2C and weight-group (z = 1.73, p = .083) and body satisfaction (z = -1.58, p = .114) did not differ significantly between the older and the younger group. However, it is worth noting that the correlation between positive body image and physical appearance self-esteem was higher for the older group compared to the younger group (z = -3.47, p < .01). Additionally, the correlation between body appreciation and global self-esteem was also higher in the older group (z = -2.78, p < .05) as well as the correlation between positive body image and BMI (z = -5.62, p < .01).

**Table 6:** Descriptive statistics and correlations (Spearman’s rho) between body appreciation and other measures included in the study for younger group (upper diagonal) and older group (bottom diagonal).

Variables	Younger group M(SD)	Older group M(SD)	[1]	[2]	[3]	[4]	[5]	[6]
[1] Body appreciation	42.49 (6.68)	37.42 (8.86)	--	-.17*	-.16*	.57**	.56**	.24**
[2] BMI	16.70 (2.52)	19.54 (3.84)	-.44**	--	.54**	-.36**	-.20**	-.41**
[3] Weight-group ^a^	.14 (.60)	.41 (.71)	-.16*	.54**	--	-.26**	-.12	-.32**
[4] Physical appearance self-esteem	3.27 (.75)	2.75 (.87)	.82**	-.46**	-.26**	--	.60**	.42**
[5] Global self-esteem	3.45 (.56)	2.92 (.75)	.78**	-.33**	-.12	.81**	--	.26**
[6] Body satisfaction	-.62 (1.11)	-.65 (1.13)	.48**	-.72**	-.32**	.47**	.46**	--

* p < .05

** p < .01

^a^ Kendall’s tau correlation

## Discussion

The aim of this study was to investigate the psychometric properties of the Polish version of the Body Appreciation Scale—2 for children aged 8–13 years, thereby expanding the age range beyond that of the groups of children participating in previous studies [13, 14, 21]. CFA analysis confirmed that the Polish version of BAS-2C has a one-factor structure, consistent with other studies [13, 14, 21]. The BAS-2C was invariant across gender which is consistent with the Japanese version of the scale [14] but distinct to Portuguese [13] study. Our invariance analysis exhibited also invariance in the age groups. The model fit in the current study was good even without adding any modification indices. However, adding correlation of the error covariances between items #1 and #9 resulted in significant improvement of the model fit, similarly as in previous studies on various other language versions of the scale for children, adolescents and adults [13, 53, 54]. Modification of one word in item 9 from "comfortable" to "good” could cause semantic convergence of items 1 and 9 in the Polish version, supporting the hypothesis of Portuguese researchers that linguistic differences can bring these items semantically closer in some language versions of the BAS-2C [13].

The results of internal consistency and test-retest reliability demonstrated that our scale has excellent internal consistency reliability and time stability. The test-retest results are similar to results from England and Japan. The internal consistency results are also consistent with findings on English, Japanese and Portuguese versions of BAS-2C [13, 14, 21].

Overall, our results support very good construct validity of the Polish version of BAS-2C. First, we found that positive body image correlates negatively with the desire to have a thinner body. According to Tylka and Wood-Barcalow [2], positive body image and body dissatisfaction are distinct but negatively correlated body image factors, which is in line with our results and also the previous findings of other researchers [4, 13, 25]. In addition, positive body image had a negative association with BMI and weight group. Among boys, those with normal classification reported higher body satisfaction than those who were obese. On the other hand, underweight and normal-weight girls expressed more body satisfaction than girls who were classified as overweight and obese (see S2 Appendix). Current beauty ideals in Western culture [7, 55, 56] favor thinnes for females and muscularity for males, with societal norms emphasizing focus on beauty and self-care more for women than for men [7]. Being overweight and obese is not only undesired, but also perceived as a lack of self-maintenance. Furthermore, research indicates that overweight or obese children often experience weight-related bullying and teasing, making them prone to weight discrimination and physical, verbal, and relational peer violence [57–59] fostering a cycle of low self-esteem and negative body image [26] which are risk factors of eating disorders, depression, anxiety disorders and chronic stress [8, 60].

Our findings also indicate that positive body image is positively correlated with both physical appearance and global self-esteem subscales which is consistent with previous research suggesting an association between self-esteem and body image [9, 10]. Additionally, our results pointed out that there is a stronger correlation between positive body image and physical appearance and global self-esteem in children aged 11–13 years than in children aged 8–10 years. The older group consisted of the children entering into adolescence, a period which is particularly vulnerable for the development of body dissatisfaction [61, 62]. As the body image is an important part of self-concept, it could be a reason why both physical appearance and self-esteem show a stronger correlation with positive body image in this age group. A study conducted by Levine and Smolak [63] have shown that body image, in conjunction with general appearance, is one of the most important elements of an adolescent’s self-esteem. Additionally, we found significant differences in correlation coefficients only between positive body image and self-esteem in boys and girls with a stronger correlation observed for girls. Girls might be more inclined to internalize the social expectations of ideal body image than boys given the heightened emphasis on appearance placed on females [7]. For this reason, girls might regularly focus on their appearance and its perception by other people. Consequently, they may frequently evaluate themselves based on how they believe their bodies and overall appearance are judged by others, making it difficult to separate their physical appearance from the intrinsic qualities and competencies.

Moreover, we found that age is negatively correlated with positive body image. The result is similar to previous findings in children [30, 64] showing that older children had lower levels of positive body image, so dissatisfaction with the body increases with age [65]. Furthermore, body changes during adolescent development can be unpredictable and sometimes undesirable, causing varying reactions from children. As children mature and separate from their parents, the influence of peers, internet, and media promoting ideal body images intensifies, along with a desire to fit in and conform to these ideals for social acceptance, contributing to body image challenges [7, 12, 65].

Furthermore, we are the first ones to attempt to use the scale for younger and older children beyond its intended scope by the authors. In our view, it is important to expand the age range for scale usage in order to investigate trajectories and dynamics of positive body development in children. This approach allows to investigate when positive body image declines in children, informing timely interventions. It also enables the identification of risk and protective factors in younger children and facilitates longitudinal studies on the trajectories of development of positive body image and the effectiveness of interventions. Additionally, we see the potential for this scale to be used not only in scientific research. In clinical settings, the BAS-2C can be integrated into psychological assessments to facilitate early identification of body image issues in children and adolescents. Even though the scale does not have specific cutoffs or established norms and thus should be used with caution in the clinical settings, it can provide valuable information for the clinicians. Clinicians can use the BAS-2C to monitor progress throughout therapy, tailoring interventions to the specific needs of each child based on their responses. The scale can serve also as a starting point for conversations about appearance and body perception in children in various age groups. In addition, the Body Apperception Scale for Children can be used to measure the effectiveness of workshops and psychoeducation in shaping children’s positive body image and making necessary adjustments to enhance their effectiveness. This can be accomplished by measuring children’s positive body image before and after the intervention. Moreover, our research indicates a significant need for these early interventions among school-aged children, as positive body image correlates positively with self-esteem, and negatively with age. Thus, it is important to foster and protect the development of a positive body image at a young age. Previous studies show that [66–68] workshops and psychoeducation can be helpful in shaping positive body image, particularly for girls, but also for boys. Based on our results and findings from previous studies [13, 14, 21] it can be anticipated that fostering positive body image could increase self-esteem and positive body attitudes as children mature, potentially mitigating the development of severe psychological conditions such as eating, depressive, and anxiety disorders.

The presented study also had its limitations. Although it was conducted in different schools among children from families with various socioeconomic status, the sample of participants was not representative of the entire population of 8-13-year-old children from Poland. Specifically, our sample was drawn exclusively from a large city in Poland, which may limit the generalizability of the findings to other regions, including rural areas and smaller towns. While not essential for validation research, conducting the study with a more representative Polish sample could both deepen our understanding of psychometric properties of the BAS2-C and make its findings more generalizable for this age group in Poland. Our participant count is sufficient for conducting all validation analyses, albeit smaller in comparison to other validation studies of BAS-2C [13, 14]. Nevertheless, the validation outcomes demonstrate excellent psychometric properties of BAS2-C. Additionally, the younger group outnumbers the older group of children. Although appropriate statistical analysis has been conducted to address this disproportion, this disparity might have influenced the results of the age-related analyses. The smaller sample size in the older group could lead to less reliable estimates and lower statistical power for detecting differences or correlations specific to this group. Increasing the number of the older children would enhance the statistical power leading to more reliable and robust results. As there is a lack of other validated tools measuring diverse body image dimensions in children in Poland, we used only the Figures Rating Scale [18] to measure body satisfaction in order to confirm the validity of BAS - 2C. Nonetheless, we have augmented our validation analyses by incorporating BMI, weight categories, physical appearance and global self-esteem. Furthermore, we obtained height and weight data from parents without the involvement of a specialized researcher. Consequently, the lack of precision in these data may result in less accurate information regarding Body Mass Index (BMI) and weight categorization. Our sample included children from each weight category, but the subgroup sizes of children in non-normal weight categories were insufficient for meaningful intergroup comparisons. Apart from gathering test-retest reliability data, our study employed a cross-sectional design, which meant it could not investigate dynamic changes in positive body image over time across children’s development.

## Conclusions

Our study extends the knowledge by providing a validated and reliable tool—the Polish version of the Body Appreciation Scale-2 for children, to measure positive body image in children. We demonstrated its very good psychometric properties, making it a viable tool for research studies in Poland. Our study also demonstrated that body appreciation is positively associated with younger age, lower BMI, physical appearance and global self-esteem. Moreover, our research demonstrated that among older children the correlation between positive body image and global self-esteem is higher than in a younger group. It lays a foundation for future research and planning interventions. This paves the way for devising strategies, like psychological support and educational programs, aimed at promoting positive body image, preventing weight-related bullying, and enhancing mental health among school-aged children. The previous findings suggest that early interventions, possibly within elementary school settings as proposed by Smolak [28], could be helpful in fostering a positive body image, thereby contributing positively to the mental health and well-being of children.

## Supporting information

S1 Appendix Sociodemographic characteristics. (DOCX)

S2 Appendix Differences between weight groups among boys and girls. (DOCX)
