# Supplementary material for: Psychometric evaluation of the Polish adaptation of the Body Appreciation Scale-2 for Children (BAS-2C)
Source: PLoS One. 2024 Sep 12;19(9):e0309945. doi: 10.1371/journal.pone.0309945 (PMC11392422; doi:10.1371/journal.pone.0309945)
Supplement: S1 Appendix — (DOCX) [file pone.0309945.s001.docx]

**Appendix S1. Sociodemographic characteristics**

Table S1. Sociodemographic characteristics

| Variables | | N = 206 | |
| --- | --- | --- | --- |
|  |  |  | |
|  |  | M | SD |
| Age (in years) |  | 9.95 | 1.45 |
|  |  | N | Percent |
| Gender |  |  |  |
|  | female | 106 | 51.5% |
|  | male | 100 | 48.5% |
| Parental situation |  |  |  |
|  | Raised by both parents | 177 | 85.9% |
|  | Raised only by mother | 11 | 5.5% |
|  | Raised only by father | 1 | .5% |
|  | Raised by legal guardian | 1 | .5% |
|  | Raised by one of parent and his partner | 9 | 4.4% |
|  | Decline to answer | 4 | 2% |
|  |  |  |  |
| Financial situation |  |  |  |
|  | Bad | 5 | 2.4% |
|  | Rather bad | 32 | 13.6% |
|  | Average | 75 | 35.4% |
|  | Rather good | 52 | 29.1% |
|  | Very good | 16 | 9.2% |
|  | Decline to answer | 23 | 10.2% |
| Mother’s Education |  |  |  |
|  | Primary education | 4 | 1.9% |
|  | Secondary education | 29 | 14.1% |
|  | Higher education | 160 | 77% |
|  | Academic Degree | 6 | 2.9% |
|  | Decline to answer | 7 | 3.4% |
| Father’s Education |  |  |  |
|  | Primary education | 9 | 5.5% |
|  | Secondary education | 53 | 27% |
|  | Higher education | 123 | 56.5% |
|  | Academic Degree | 13 | 8% |
|  | Decline to answer | 8 | 3.9% |

**Supporting Information S2. Differences between weight groups among boys and girls**

Table S2. Differences between weight groups among boys and girls

|  | Kruskal–Wallis test | | Multiple comparisons test (*p*-values) | | | | | | |
| --- | --- | --- | --- | --- | --- | --- | --- | --- | --- |
|  | *H* | *p* | obesity- overweight | obesity- underweight | obesity-  norm | overweight- underweight | | overweight- norm | underweight- norm |
| boys  (n = 100) | 8.305 | .040 | .102 | .256 | .004 | | .860 | .446 | .866 |
| girls  (n=106) | 17.907 | <.001 | .668 | .007 | .005 | | .004 | .003 | .292 |
| total sample  (N = 206) | 23.221 | <.001 | .363 | .003 | <.001 | | .022 | .007 | .401 |
