# Supplementary material for: Psychometric evaluation of the Polish adaptation of the Body Appreciation Scale-2 for Children (BAS-2C)
Source: PLoS One. 2024 Sep 12;19(9):e0309945. doi: 10.1371/journal.pone.0309945 (PMC11392422; doi:10.1371/journal.pone.0309945)
Supplement: S2 Appendix — (DOCX) [file pone.0309945.s002.docx]

**Appendix S2. Differences between weight groups among boys and girls**

Table S2. Differences between weight groups among boys and girls

|  | Kruskal–Wallis test | | Multiple comparisons test (*p*-values) | | | | | | |
| --- | --- | --- | --- | --- | --- | --- | --- | --- | --- |
|  | *H* | *p* | obesity- overweight | obesity- underweight | obesity-  norm | overweight- underweight | | overweight- norm | underweight- norm |
| boys  (n = 100) | 8.305 | .040 | .102 | .256 | .004 | | .860 | .446 | .866 |
| girls  (n=106) | 17.907 | <.001 | .668 | .007 | .005 | | .004 | .003 | .292 |
| total sample  (N = 206) | 23.221 | <.001 | .363 | .003 | <.001 | | .022 | .007 | .401 |
